# Dopamine Modulates Metabolic Rate and Temperature Sensitivity in *Drosophila melanogaster*

**DOI:** 10.1371/journal.pone.0031513

**Published:** 2012-02-07

**Authors:** Taro Ueno, Jun Tomita, Shoen Kume, Kazuhiko Kume

**Affiliations:** 1 Department of Stem Cell Biology, Institute of Embryology and Genetics, Kumamoto University, Kumamoto, Japan; 2 Global COE program, Kumamoto University, Kumamoto, Japan; Yale School of Medicine, United States of America

## Abstract

Homeothermal animals, such as mammals, maintain their body temperature by heat generation and heat dissipation, while poikilothermal animals, such as insects, accomplish it by relocating to an environment of their favored temperature. Catecholamines are known to regulate thermogenesis and metabolic rate in mammals, but their roles in other animals are poorly understood. The fruit fly, *Drosophila melanogaster*, has been used as a model system for the genetic studies of temperature preference behavior. Here, we demonstrate that metabolic rate and temperature sensitivity of some temperature sensitive behaviors are regulated by dopamine in *Drosophila*. Temperature-sensitive molecules like dTrpA1 and *shi* ^ts^ induce temperature-dependent behavioral changes, and the temperature at which the changes are induced were lowered in the dopamine transporter-defective mutant, *fumin*. The mutant also displays a preference for lower temperatures. This thermophobic phenotype was rescued by the genetic recovery of the dopamine transporter in dopamine neurons. Flies fed with a dopamine biosynthesis inhibitor (3-iodo-L-tyrosine), which diminishes dopamine signaling, exhibited preference for a higher temperature. Furthermore, we found that the metabolic rate is up-regulated in the *fumin* mutant. Taken together, dopamine has functions in the temperature sensitivity of behavioral changes and metabolic rate regulation in *Drosophila*, as well as its previously reported functions in arousal/sleep regulation.

## Introduction

Almost all animals physiologically adjust their energy consumption rate in response to a wide range of environmental changes, including ambient temperature. In order to maintain their proper body temperature, they need more energy at lower temperatures, with the exception of hibernating animals. Mobile animals actively select and relocate to a place of their preferred temperature so that they can maintain their body temperature with less energy expenditure [1]. Thus, in order to understand the mechanisms of energy balance, studies of behavior as well as physiological activity at the organ and cellular levels are required.

Studies on thermotaxis and temperature preference behavior have been performed in model organisms, including the fruit fly *Drosophila melanogaster* (reviewed in Dillon et al. [2]) and *Caenorhabditis elegans* [3], [4], [5]. However, the molecular mechanisms mediating metabolic regulation and temperature preference behavior remain elusive. In *Drosophila*, several genes related to temperature sensing have been characterized, including *dTRPA1*, *Painless*, *Hsp70*, *Pyrexia*, and *brivido* [6], [7], [8], [9], [10]. The biogenic amine histamine was reported to modulate temperature preference behavior in *Drosophila* [11].

The role of catecholamines on energy balance has been studied in mammals. Mice lacking both noradrenaline and adrenaline showed impaired thermogenesis and metabolic regulation [12]. Catecholamine reuptake inhibitors, such as bupropion, promoted thermogenesis and resulted in weight loss [13].

We have employed *Drosophila melanogaster* as a model animal for genetic studies of behaviors including sleep and arousal. We previously reported that a dopamine transporter (DAT)-defective mutant, *fumin (fmn)*, showed a short sleep phenotype, identifying dopamine's function in sleep and arousal regulation in *Drosophila* – a function conserved also in mammals [14], [15]. During further analysis of dopaminergic sleep regulation using genetic behavioral assays based on temperature-dependent molecules, we found that the temperature sensitivity of the *fmn* mutant is different from that of the wild type flies. That means we found that the temperatures at which *fmn* mutants showed behavioral changes were significantly lower. In this study, we show that the dopaminergic system participates in regulating metabolic rate and temperature sensitivities of some temperature-dependent behavioral changes, as well as modulating temperature preference in *Drosophila*. Elevated dopamine signaling in the *fmn* mutants resulted in an increased metabolic rate and a thermophobic phenotype – a preference for lower temperatures compared to wild type flies. These results highlight similarities between insects and mammals in the molecular basis of energy homeostasis.

## Results

### Increased temperature sensitivity of dTrpA1 and *shi* ^ts^ in *fmn* mutants

**Figure 1:**
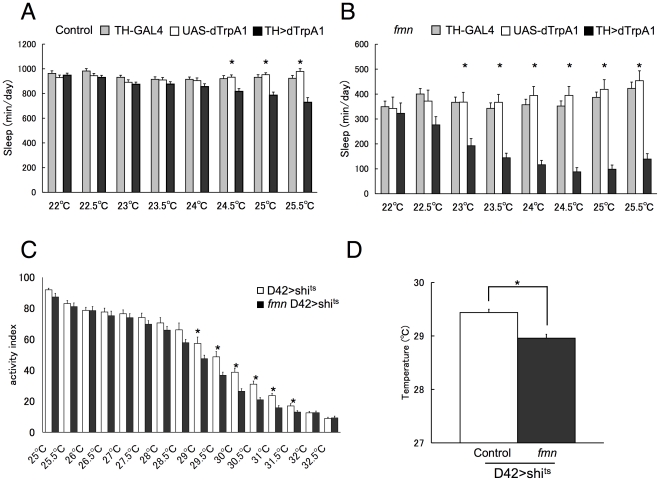
*fmn* mutant flies expressing dTrpA1 or *shi* ^ts^ showed increased temperature sensitivity compared to control flies. A. Sleep time of control flies with TH-GAL4, UAS-TrpA1, or both at different ambient temperatures. B. Sleep time of *fmn* flies with TH-GAL4, UAS-TrpA1, or both at different ambient temperatures. Data are represented as mean ± SEM (n = 17–32 flies). Asterisks indicate that TH-dTrpA1 flies showed statistically significant differences from TH-GAL4 or UAS-dTrpA1 flies. Statistical significance was determined by one-way ANOVA with Tukey-Kramer HSD *post hoc* test for normally distributed data (*p*<0.05). C. Activity changes of control (white bar) and *fmn* flies (black) expressing D42>*shi* ^ts^ at elevated temperatures. n = 10 experiments with approximately 20 flies. D. Temperature at which the activity index decreased to 50% of that at 25°C calculated from C. Data are represented as mean ± SEM. Asterisk indicates a statistically significant difference (Student's *t*-test; *p*<0.05).

*fmn* flies carry a mutation in the *Drosophila DAT* (d*DAT*) gene as previously reported [14]. The dDAT protein has significant sequence similarity to mammalian DAT and its expression is restricted to dopaminergic neurons (as expected for a presynaptic transporter). dDAT also has a substrate specificity paralleling that of mammalian DATs, with dopamine and tyramine being the preferred substrates [16]. Dopamine is cleared from the synaptic cleft via presynaptic DAT, indicating that postsynaptic dopamine signaling in *fmn* mutants is likely to be increased [17]. To further study the contribution of dopamine in sleep regulation, we use GAL4-UAS binary system, in which yeast transcriptional activator, GAL4 expressed with a tissue specific promoter activates a target gene under control of UAS, a regulatory upstream activating sequence [18]. The temperature-sensitive channel dTrpA1 was expressed in dopamine neurons using TH (tyrosine hydroxylase)-GAL4 and UAS-dTrpA1. dTrpA1 is known to activate neuronal activity around 25°C [19] and as expected, control flies expressing the dTrpA1 channel in dopamine neurons showed significantly shorter sleep at temperatures above 24.5°C (Fig. 1A). However, *fmn* flies expressing the dTrpA1 channel showed significant sleep loss at temperatures above 23°C (Fig. 1B). In order to determine whether the difference in temperature sensitivity is limited to either dopamine neurons or dTrpA1, another temperature sensitive molecule, temperature sensitive dominant negative dynamin, *shibire* ^ts^ (*shi* ^ts^) [20], was expressed in motor neurons with the D42-GAL4 driver [21]. We monitored locomotor activity of flies under controlled temperatures. At elevated temperatures, flies expressing *shi* ^ts^ in motor neurons showed paralysis (Fig. 1C, Movie S1). *fmn* mutants expressing *shi* ^ts^ in motor neurons showed significantly more severe paralysis compared to control flies at the same temperature (Fig. 1C). The temperature at which the activity index decreased to 50% of that at 25°C was significantly lower in *fmn* flies (Fig. 1D). To further investigate the effect of dopamine signaling, we next tested the temperature preference behavior.

### DAT mutants *fmn* show preference for lower temperature

**Figure 2:**
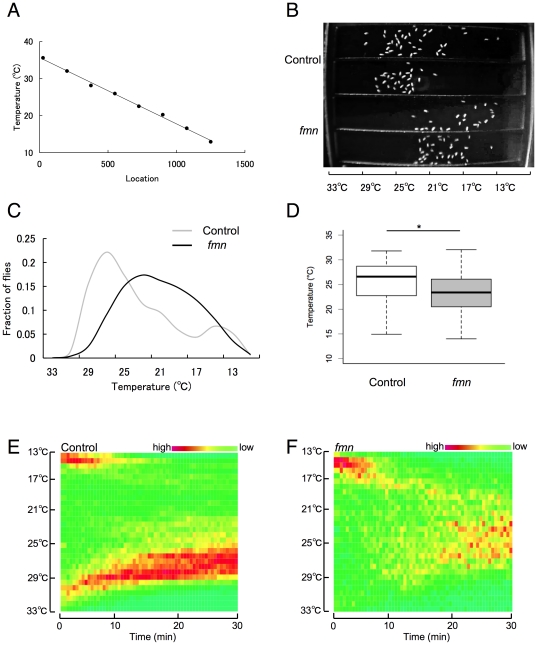
*fmn* mutant flies show a preference for lower temperatures than control flies. A. Temperature gradient of the apparatus. Actual temperature readout from thermocouples placed under the aluminum chamber indicating that a linear temperature gradient from 10 to 35°C was established. The temperatures were monitored simultaneously during the assay. B. Picture of a temperature preference assay after 25 min exposure to thermal gradient. Flies are reflected as white dots. Upper two lanes: control flies. Lower two lanes: *fmn* flies. Temperatures of horizontal positions are indicated. C. Quantified results showing the distributions of flies along the thermal gradient after 25 min exposure to thermal gradient. D. Preferred temperature calculated from the location of the control (*n* = 599) and *fmn* (*n* = 546) flies after 25 min exposure to thermal gradient. In each box plot, the box encompasses the interquartile range, a line represents the median, and the error bars encompass the 10th and 90th percentiles. Asterisk indicates a statistically significant difference (Mann–Whitney U test; *p*<0.001). E. Dynamics of temperature preference in control flies. F. Dynamics of temperature preference in *fmn* flies.

The temperature preference of adult *Drosophila* was examined on a linear temperature gradient apparatus. Fig. 2A shows a temperature gradient from 10 to 35°C maintained in the apparatus. Approximately 30 flies were transferred from a culture vial to each lane of the gradient through holes in the cover. When flies were allowed to distribute along a thermal gradient for 25 min, control adults preferred ∼25°C (Fig. 2B, C) as previously reported [3], and the mean preference temperature for the population was 25.29°C (Fig. 2D). Compared to controls, *fmn* mutants displayed a thermophobic phenotype (Fig. 2B, C). The mean preference temperature for the *fmn* mutant was 23.27°C (Fig. 2D). Furthermore, the temporal dynamic analysis of the spatial distribution revealed that control flies preferred higher temperature at the first half of the assay and then gradually shifted to lower temperature (Fig. 2E). On the other hand, *fmn* mutants approached to their prefered temeprature from lower temeperature (Fig. 2F).

### DAT expression in dopamine neurons restores preference for lower temperatures in *fmn* mutants

**Figure 3:**
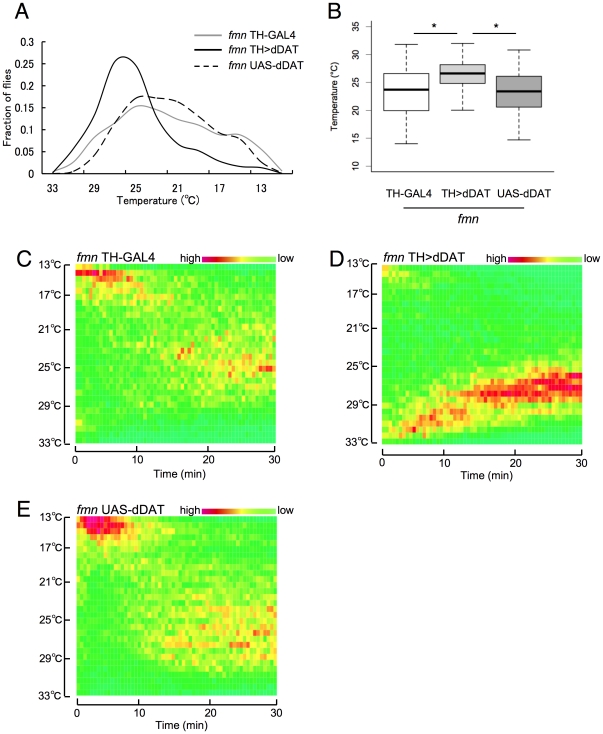
dDAT expression in dopamine neurons rescues low-temperature preference phenotype of *fmn* mutants. A. Distribution of flies along a thermal gradient after 25 min exposure to thermal gradient. B. Preferred temperature of *fmn* mutants; *fmn* ; TH-GAL4 (*n* = 230), *fmn* UAS-dDAT ; TH-GAL4 (*n* = 242) and *fmn* UAS-dDAT (*n* = 245) after 25 min exposure to thermal gradient. In each box plot, the box encompasses the interquartile range, a line represents the median, and the error bars encompass the 10th and 90th percentiles. Asterisk indicates a statistically significant difference (Kruskal-Wallis test; *p*<0.001) C. Dynamics of temperature preference in *fmn* ; TH-GAL4. D. Dynamics of temperature preference in *fmn* UAS-dDAT ; TH-GAL4. E. Dynamics of temperature preference in *fmn* UAS-dDAT.

To confirm whether the altered temperature preference exhibited in *fmn* mutants was caused by the loss of function of dDAT in dopamine neurons, we next looked at the rescue of *fmn* by dDAT cDNA expression using transgenic flies. Two transgenic fly lines in an *fmn* background, each homozygous for UAS-dDAT and TH-GAL4 transgenes, respectively, were crossed. The resulting double transgenic flies were heterozygous for both transgenes, and in these flies the UAS-dDAT transgene was driven under the control of TH-GAL4. These flies showed temperature preferences that were restored to normal, while the parental single transgenic flies showed the same thermophobic phenotype as *fmn* mutants (Fig. 3A,B). The dynamics of temperature preference behavior were also restored in *fmn* TH>dDAT flies. These flies showed the gradual movement to their preferred temperature from the high temperature side (Fig. 3D), while the parental single transgenic flies approached to their preferred temperature from the lower temperature side (Fig. 3C,E).

### Dopamine biosynthesis inhibitor altered temperature preference

**Figure 4:**
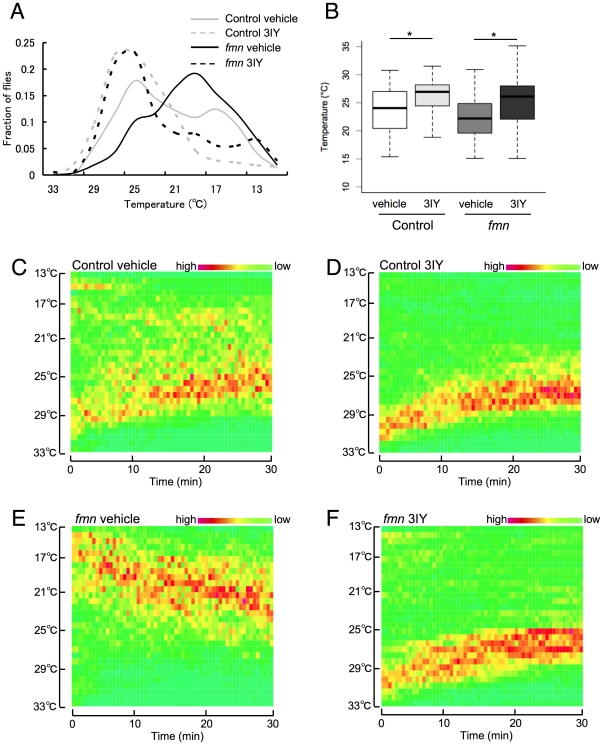
The dopamine biosynthesis inhibitor, 3-iodo-L-tyrosine, raised the preferred temperature both in control and *fmn* flies. A. Distribution of flies along a thermal gradient after 25 min exposure to thermal gradient. B. Preferred temperature of control flies fed with vehicle (*n* = 203), control flies fed with 3IY (*n* = 207), *fmn* flies fed with vehicle (*n* = 201) and *fmn* mutants fed with 3IY (*n* = 187) after 25 min exposure to thermal gradient. In each box plot, the box encompasses the interquartile range, a line represents the median, and the error bars encompass the 10th and 90th percentiles. Asterisk indicates a statistically significant difference (Kruskal-Wallis test; *p*<0.001) C. Dynamics of temperature preference in control flies fed with vehicle. D. Dynamics of temperature preference in control flies fed with 3IY. E. Dynamics of temperature preference in *fmn* flies fed with vehicle. F. Dynamics of temperature preference in *fmn* flies fed with 3IY.

To assess whether dopamine signaling regulates temperature preference, the effect of a dopamine biosynthesis inhibitor was investigated. Both control and *fmn* mutant flies were fed with 3 mM 3-iodo-L-tyrosine (3IY) for 48 h and their temperature preferences were determined. The drug inhibits the activity of tyrosine hydroxylase, a rate-limiting enzyme in dopamine biosynthesis, and significantly decreases steady-state amounts of dopamine after 2 days of feeding [22]. We used 1% agar with 5% sucrose as a vehicle, and found that both control and *fmn* flies fed with 1% agar with 5% sucrose showed a preference for lower temperatures compared to flies fed with standard food (control flies: 25.29°C/23.83°C, standard food/vehicle, *fmn* mutants: 23.27°C/22.21°C) (Fig. 2D and 4B). *fmn* mutants consistently preferred lower temperatures compared to control flies when fed with 1% agar with 5% sucrose. The administration of 3IY significantly increased the preferred temperature both in control flies and *fmn* mutants (Fig. 4A, B). The dynamics of temperature preference behavior were also restored with 3IY administration in *fmn* flies (Fig. 4C–F). These results indicated that the temperature preference is modulated by dopamine signaling in flies and that their changes in the *fmn* mutants are the result of altered dopamine signaling.

### Increased metabolic rate in *fmn* mutants

**Figure 5:**
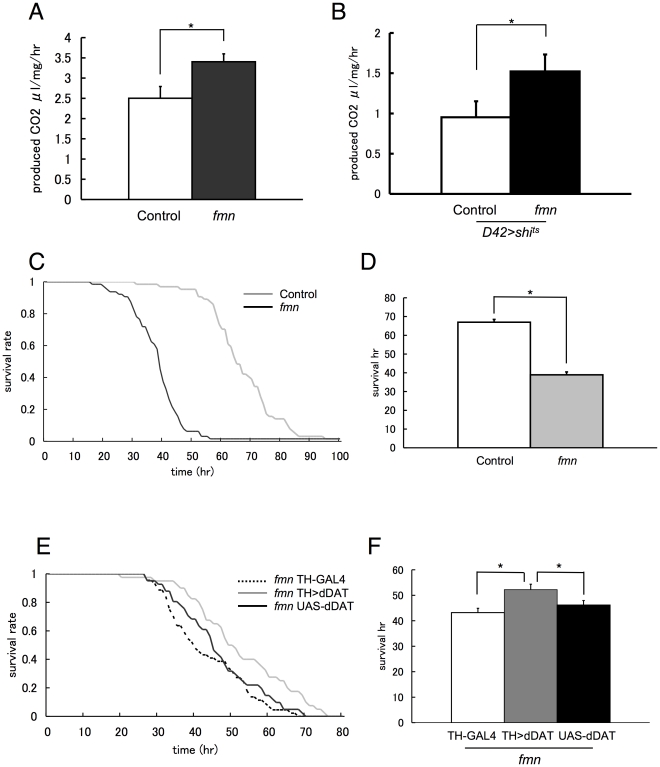
*fmn* mutants showed increased energy metabolism due to enhanced dopamine signaling. A. Metabolic rates as measured by CO_2_ production (*n* = 12 for each genotypes). Data are represented as mean ± SEM. Asterisks indicate statistically significant differences (Student's *t*-test; *p*<0.05). B. Metabolic rates as measured by CO_2_ production without locomotion (*n* = 8 for each genotypes). Data are represented as mean ± SEM. Asterisks indicate statistically significant differences (Student's *t*-test; *p*<0.05). C. Survival curves of control and *fmn* flies on starvation (*n* = 64 for each genotype). D. Mean survival time of control and *fmn* flies during starvation. Data are represented as mean ± SEM. Asterisks indicate statistically significant differences (Student's *t*-test; *p*<0.05). E. Survival curves of *fmn*; TH-GAL4, *fmn* UAS-dDAT; TH-GAL4, and *fmn* UAS-dDAT flies on starvation (*n* = 64 for each genotype). F. Mean survival time of *fmn* ; TH-GAL4, *fmn* UAS-dDAT ; TH-GAL4, and *fmn* UAS-dDAT flies during starvation. Data are represented as mean ± SEM. Asterisks indicate statistically significant differences (one-way ANOVA followed by the Tukey-Kramer *post hoc* test; *p*<0.05).

Many ectothermic animals compensate for thermal changes by altering their metabolic rate, and the acclimation to low temperatures is frequently associated with an increased metabolic rate [23]. In *Drosophila*, an increased metabolic rate and preference for lower temperatures in the dystroglycan mutant, *atsugari*, has been reported [24]. Therefore, we examined whether *fmn* mutants have an altered metabolic rate. In *Drosophila*, the production of CO_2_ is correlated directly with oxygen consumption and reflects the metabolic rate with absolute accuracy [25]. Thus we measured the amount of CO_2_ produced by *Drosophila* using respirometry. As shown in Fig. 5A, the metabolic rate of the *fmn* mutants measured by respirometry was significantly increased compared with control flies (Fig. 5A). Since *fmn* mutants show short sleep phenotype, increased CO_2_ production in *fmn* could be the secondary effect of their increased locomotor activity. To exclude this possibility, we expressed *shi* ^ts^ in motor neurons by D42-GAL4 driver. As shown in Fig. 1C, *shi* ^ts^ shows an equivalent or higher effect when expressed in *fmn* compared when it is expressed in control. The metabolic rate was measured at 30°C where both control and *fmn* stop moving by the effect of *shi* ^ts^. We confirmed that the locomotion of flies were ceased with direct observation. Although the CO_2_ production were decreased without fly locomotion, the metabolic rate of the *fmn* mutants was significantly higher compared with control flies (Fig. 5B). For further validation of increased energy expenditure in *fmn* mutants, we evaluated their resistance to starvation. The average survival time for food-deprived flies was determined using the Trikinetics *Drosophila* activity monitoring system. As expected, we found that flies with disrupted dDAT were more sensitive to starvation than control flies (Fig. 5C,D). Expression of dDAT with the TH-GAL4 driver moderately but significantly rescued the sensitivity to the starvation (Fig. 5E,F).

### Cold resistance of *fmn* mutants

**Figure 6:**
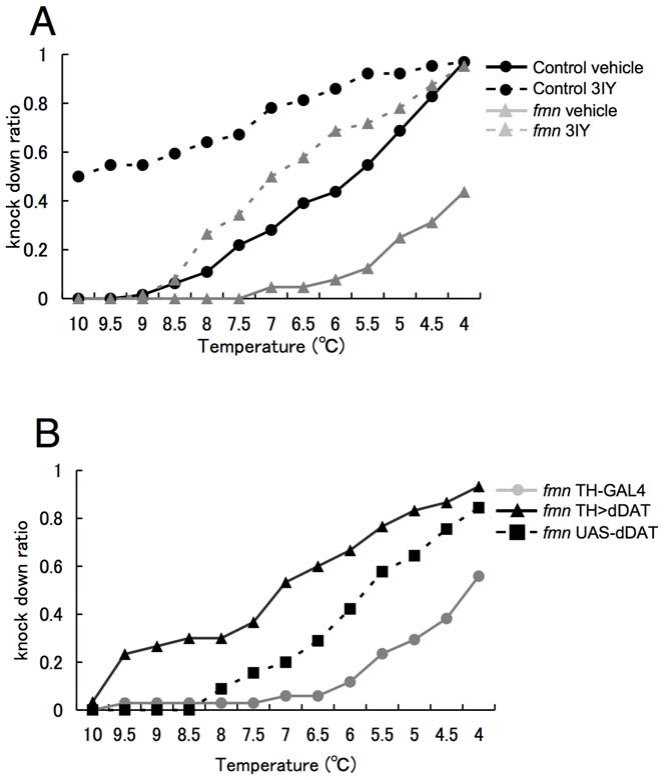
*fmn* mutants showed increased cold tolerance due to enhanced dopamine signaling. A. Resistance to cold stress, which was tested by DAM assay with decreasing temperature. Solid line indicates flies fed with vehicle, dashed line indicates flies fed with 3IY. (n = 61–64) B. Resistance to cold stress with genetic rescue. (n = 39–45).

To further address the relationship between metabolic rate and dopamine signaling, we examined the changes in the tolerance against low temperature, which is an indicator for critical thermal limits [26],. When control flies fed with vehicle (1% agar +5% sucrose) are exposed to gradually lowering temperature, they started to be knocked-down when the temperature became 9°C and all flies were knocked-down at 4°C (Fig. 6A). On the other hand, *fmn* mutants showed resistance to lower temperature and half of them could still move at 4°C. Administration of 3IY reversed the tolerance to lower temperature in *fmn* mutants to that of control. Genetic rescue of *fmn* mutants with TH-GAL4/UAS-dDAT also restored the preference to lower temperature (Fig. 6B). Collectively, these results suggested that the enhanced dopamine signaling results in tolerance to low temperature, or the decrease in the lower thermal limit.

## Discussion

In flies, dopamine has already been reported to be involved in development [22], aversive olfactory associative memory formation [27], decision making [28], and arousal [14], [29]. This study is the first to show that dopamine is involved in both metabolic rate and temperature sensitivities of some temperature dependent behaviors in *Drosophila*.

DAT is expressed in dopamine neurons [16] and is responsible for the reuptake of dopamine from the synaptic cleft [17] keeping extracellular dopamine concentrations low. The lack of DAT leads to deficient dopamine reuptake, which then results in increased dopamine signaling. Our previous study showed that the DAT mutant, *fmn*, shows reduced sleep [14]. Despite the continuous excess of dopamine, the dopamine signaling pathway appeared not to be down-regulated in *fmn* mutants, since their short sleep phenotype continues almost for the whole of their life span. As we demonstrated above, the effects of temperature-sensitive effector molecules, such as dTrpA1 and *shi* ^ts^, was more evident at the lower temperature in the *fmn* background. dTrpA1 channel has a higher resting input resistance at lower temperature [30], and *shi* ^ts^ shows defective synaptic vesicle recycling only at the restrictive temperature [20]. One possible explanation for the higher temperature sensitivity is that the increased endogenous activity of dopamine neurons in *fmn* mutants could have activated these effector molecules in temperature-independent way. For the study of biophysical properties of temperature-sensitive molecules, the study with cold-activated molecule such as TRPM8 may be informative [31]. However, our further analysis raised the possibility that the changes in temperature preference behaviors in *fmn* mutants might be due to increased metabolic rate. *fmn* mutants showed a preference for lower temperatures compared to the wild type, and their metabolic rate was also up-regulated. It is also possible that increased metabolism resulted in the altered temperature sensitivities in the other temperature dependent behaviors, although further studies are necessary to prove the causal relationship between these changes. Taken together, these results indicate that dopamine plays a role in temperature related behaviroral changes as well as sleep regulation in *Drosophila*. Some insects like moths, dragonflies, bumblebees and honeybees can maintain thermal stability by regulating heat gain and heat loss [32]. Thermal stabilization in many insect species involves regulation of heat loss by varying hemolymph flow, enabling tissue-specific temperature regulation [33]. Further study is needed to understand the mechanism of thermal regulation in *D. melanogaster*, and the strategy adopted here of expressing temperature-sensitive molecules using the GAL4-UAS system might be valuable in the characterization of tissue-specific thermal regulation.

Although *fmn* mutants have a normal circadian rhythm, shown by eclosion rhythmicity, the daily activity and sleep profiles are quite different from the wild type flies since they take less sleep, especially during night. In this sense, dopamine is involved in the daily activity changes. Changes in environmental temperature are known to directly control the circadian clock [34], [35], [36]. Interestingly, daily changes in temperature preference were affected by the mutation in *Han*, a G protein-coupled receptor for the circadian clock neuropeptide PDF [37]. Since dopamine regulates sleep and arousal, these results imply that there might be an inter-relationship between thermal behavior and circadian rhythm mediated by dopamine, and our study may provide clues for the mechanism. In mammals, body temperature is tightly regulated by the circadian clock and actually used as a reliable output of the internal clock. The centers for thermoregulation and circadian clock are in the hypothalamus – specifically the preoptic area and suprachiasmatic nucleus [38]. Interestingly, these areas are also critical for sleep and arousal regulation.

**Figure 7:**
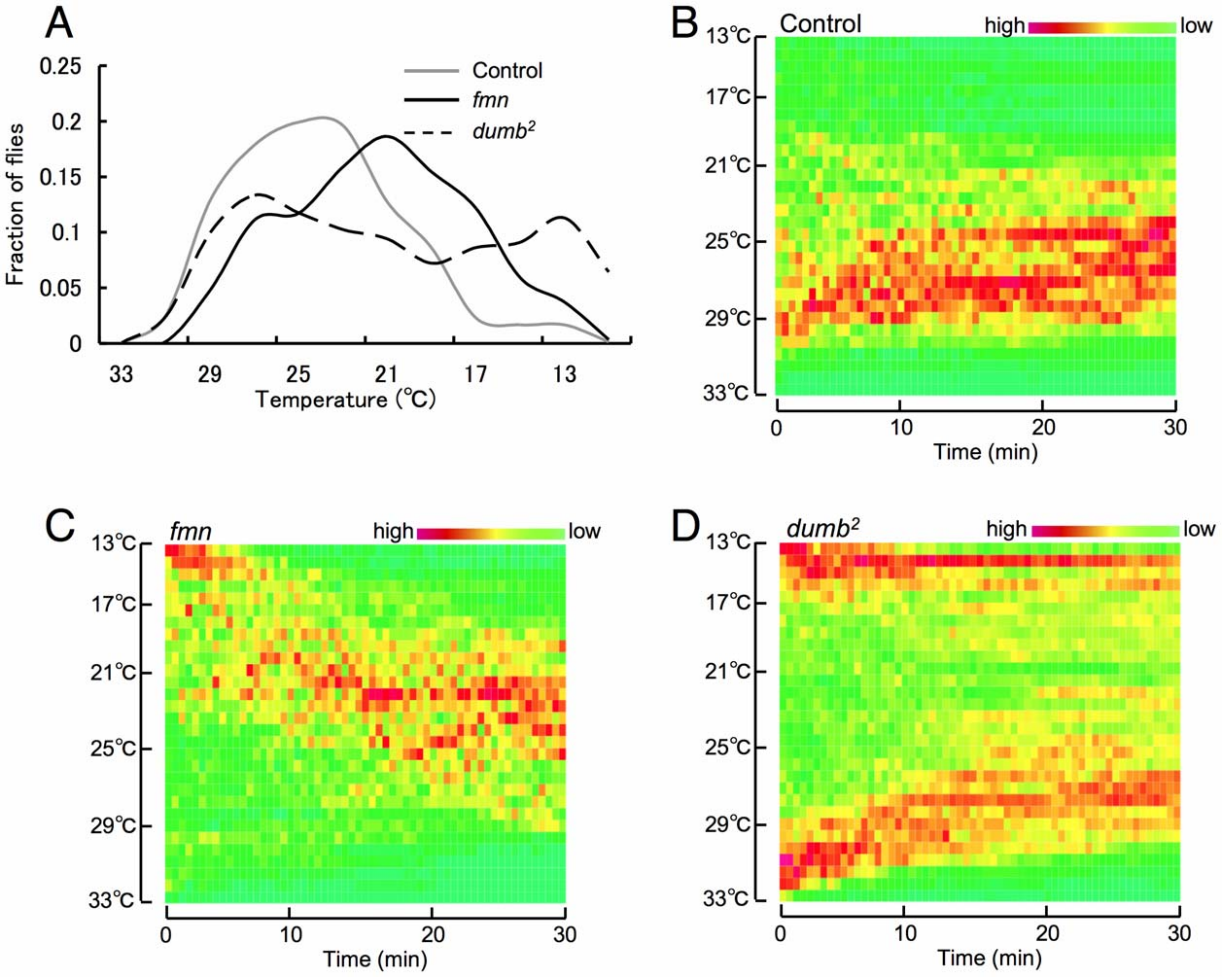
Impairment of cold avoidance in *dumb^2^* mutants. A. Distribution of flies along a thermal gradient after 25 min exposure to thermal gradient. B. Dynamics of temperature preference in control flies. C. Dynamics of temperature preference in *fmn* flies. D. Dynamics of temperature preference in *dumb^2^* flies.

In *Drosophila*, dopamine neurons are broadly distributed across the brain. Some of them innervate the neuropil of mushroom bodies and central complex [39] and it is also reported that dopamine activates protein kinase A (PKA) signaling in mushroom bodies [40]. The adult mushroom bodies have been shown to play an important role in memory consolidation [41] and sleep regulation [42], [43] and also in temperature preference regulation [44]. Importantly, cyclic AMP (cAMP)-PKA signaling in mushroom bodies regulates temperature preference behavior. Recently, Bang et al. reported that dopamine signaling in the mushroom body regulates temperature preference behavior [45] (this work was published during the preparation of this manuscript). They showed DopR mutant *dumb^3^/dumb^3^* flies spread widely over the temperature gradient lower than 25°C and the temperature preference was restored with DopR expression in mushroom body. With another DopR mutant *dumb^2^/dumb^2^*, we observed the same phenotype, as supplemented in Fig. 7. *dumb^2^/dumb^2^* flies distributed widely over lower temperature consistent with their report (Fig. 7A). However, the dynamic analysis of the spatial distribution revealed that a population of flies with *dumb^2^/dumb^2^* mutation showed a gradual movement from high temperature side to preferred temperature like control flies, and the temperature they accumulate appears rather higher than control (compare Fig. 7 B and 7D). On the other hand, another population of them stays at the low temperature side for the whole assay period (Fig. 7D), unlike *fmn* mutant shows gradual movement from cold temperature to a higher temperature, as one group (Fig. 7C). As a result, *dumb^2^/dumb^2^* mutant flies apparently split into two groups, making their distribution widely scattered without making a single peak at the preferred temperature. There is another discrepancy between their results and ours on the TH inhibitors. They described the application of 20 mM of α-methyl-p-tyrosine methyl ester (AMPT) to *pale* heterozygous mutant for 4 days resulted in cold preference like *dumb^3^/dumb^3^* DopR mutant. As described above (Fig. 4), we found 3 mM 3IY application to wild type and *fmn* mutant resulted in the preference for higher temperature. Thus, two results are apparently contrasting. However, we regard this is due to the difference both in the concentration and lenghth of the drug treatment, and fly lines used for the study. AMPT and 3IY are reported to have comparable effects on decreasing dopamine, and 5 mg/ml of them (approx. 25 mM for AMPT, and 18 mM for 3IY) almost completely depleted dopamine in fly larva [22]. In addition, our previous observations revealed that the administration of 3 mM 3IY for 2 days could rescue the short-sleep phenotype of *fmn* mutant almost to the control level (data not shown). Based on these, we regard our protocol is enough to see the physiological effect of dopamine. Four days administration of 20 mM AMPT to *pale* heterozygous mutant, which possess a lower level of dopamine than wild type control might have depleted dopamine completely, which resulted in the impairment of cold avoidance. We, therefore, propose that the increase and decrease of dopamine signaling within a physiological level, such as *fmn* mutant and the moderate inhibition of TH, result in the preference to a lower and higher temperature, respectively. On the other hand, dissipation of dopamine signaling, such as DopR mutant or prolonged administration of a TH inhibitor, results in the impairment of cold avoidance.

Recently, the distinct roles of the subset of the dopamine neurons were described. Kong et al. distinguished clusters of dopamine neurons using different GAL4 drivers (TH-GAL4, Ddc(HL5)-GAL4, Ddc(HL7)-GAL4 and Ddc(HL9)-GAL4) and found that a limited number of dopamine neurons are responsible for the promotion of ethanol-induced locomotor activity [46]. We also recently found that the activation of some clusters of dopamine neurons by Ddc(HL5)-GAL4 and Ddc(HL7)-GAL4 changed the rest interval distribution (unpublished results). The use of these GAL4 drivers in temperature preference assays may bring more insights. Further research is needed to determine the precise mechanisms and neural circuits by which dopamine regulates temperature preference behavior.

In conclusion, our study demonstrates that dopamine plays important roles in metabolic regulation and temperature sensitivity of temperature-dependent behavioral changes as well as in sleep and arousal regulations. It also provides us with new insights and a tool into the understanding of the mechanism of temperature sensing and thermotactic decisions in *Drosophila*.

## Materials and Methods

### Fly stocks

Flies were reared on a standard corn meal, yeast, glucose agar medium at 25°C under 12 h∶12 h light∶dark cycles except for the *shi* ^ts^ temperature sensitivity assay. *fmn* (*fumin*) mutants and transgenic strains for pUAS-dDAT, TH-GAL4 were described previously [14]. TH-GAL4 was a gift from J. Hirsh (Virginia Univ.). UAS-dTrpA1 was a gift from P. A. Garrity (Brandeis Univ.). D42-GAL4 and UAS-*shi* ^ts^ were from Bloomington Stock Center. To remove potential modifiers and allow comparisons in a common genetic background, we outcrossed all the alleles into the *w^1118^* background over at least 5 consecutive generations. *w^1118^* flies were used as controls.

### Temperature preference behavior

The temperature preference experiments were performed in an environmental room maintained at 17°C and 70% relative humidity. A linear thermal gradient was produced on an aluminum plate (12.5 cm×20 cm) by putting a Peltier device (90 mm×30 mm) under each longitudinal end of the plate. The device was connected to a power supply (Regulated DC supply, Sunhayato, Japan) and the voltages were empirically adjusted and fixed so that the temperature gradient was stable for an extended time. We confirmed that the temperature gradient was stable 5 min before starting each experiment. The aluminum plate was separated into four lanes (25 mm width×140 mm length) with an aluminum frame (2 mm height), and was covered by a glass plate. Eight thermocouples were placed at the back of the aluminum plate at 2-cm intervals. The temperature read-out from these thermocouples was recorded every 30 sec using a TC-08 thermocouple data logger (Pico Technology, UK) connected to a PC. Approximately 30 adult flies (0–3 days old) cultured at 25°C were placed between the aluminum and glass plates through a hole at the midpoint of the gradient without anesthesia, and exposed to the gradient during complete darkness. The chamber was illuminated with infrared LEDs and the image (256 gray scale, 1280×960 pixels) was captured every 30 sec by an infrared camera set above the chamber. The horizontal axis of the image is placed parallel with the direction of the temperature gradient, and each horizontal pixel within an image was assigned to a temperature using the actual temperature gradient information obtained from the thermocouples. Images captured were collected and the position of each fly was calculated, and assigned to a temperature using a custom-made program on a LABVIEW platform (National Instruments, TX, USA). Heat maps showing the dynamic changes of their position were made by the freely available statistical software package, R 2.11.1.(R Development Core Team). The data were further processed using Microsoft-Excel (WA, USA). The data after 25 min exposure to thermal gradient was used for statistical analysis. The statistical significance of differences was assessed as described in the figure legends using Microsoft Excel and R.

### Sleep analysis

Flies were placed individually in glass tubes (length, 65 mm; inside diameter, 3 mm) containing 1% agar and 5% sucrose at 22°C. They were entrained for at least 3 days to light∶dark conditions before transferring to constant dark (DD) conditions. Locomotor activity was monitored by recording infrared beam crossings by individual flies in 1 min bins using the *Drosophila* activity monitoring system (Trikinetics, Waltham, MA, USA). Ambient temperature was increased every day in 0.5°C steps at CT0. Data were collected continuously for 8 days under DD conditions. Based on previous reports [47], [48], sleep was defined as periods of inactivity lasting 5 min or longer. Daily sleep time was calculated with software written in R [49].

### Temperature sensitivity assay of *shi* ^ts^

Flies with D42-GAL4 and UAS-*shi* ^ts^ were crossed and reared on a standard corn meal, yeast, glucose agar medium at 20°C under a 12 h∶12 h light∶dark cycle. Flies were put on a Peltier device (60 mm×60 mm) surrounded with a black acrylic frame (2 mm height) and were covered by a glass plate. A thermocouple was placed on the Peltier device and the temperature read-out by the thermocouple was recorded every second using a TC-08 thermocouple data logger connected to a PC. The chamber was illuminated with infrared LEDs and the image (256 gray scale, 640×480 pixels) was captured by an infrared camera set above the chamber. Approximately 20 adult flies (0–3 days old) cultured at 20°C were placed directly on the Peltier device through a hole in the glass plate without anesthesia, and exposed to the experimental temperature during complete darkness. The temperature of the device was first set to 25°C by controlling the electric current based on the temperature read-out from thermocouple. The target temperature was increased in 0.5°C steps every 2 min. Image capture, temperature recording and control of the electric current to the Peltier device occurred every second with a single custom-written program on a LABVIEW platform. Another custom-made program written in LABVIEW was used for analyzing the video images. To quantify the activity of flies, we set the regions of interest (ROIs) to the area of the chamber. We subtracted 2 consecutive images pixel-wise to generate the difference images. A Gaussian filter was applied to the difference image to reduce noise. To correct for background intensity changes due to the flicker of the infrared illumination, each pixel in the difference image was binarized based on a predetermined threshold. The threshold was set to 20, where the maximum level on the grayscale was 256. This threshold value was sufficiently small compared with the difference achieved by actual fly movements, which is usually above 100. The ratio (%) of suprathreshold pixels in the total number of pixels in ROIs was used as an indicator of the degree of movement. The value was calibrated with the maximum value of the single assay and summed up in 2 min bins and defined as the activity index of the specific temperature. The statistical significance of differences among means was assessed using the Student's *t*-test.

### Drug treatment

For the pharmacological manipulations 1% agar with 5% sucrose was used as a vehicle medium. A dopamine biosynthesis inhibitor, 3-iodo-L-tyrosine (3IY, Sigma-Aldrich, MO, USA) was mixed into the medium to a final concentration of 3 mM. Flies were transferred and kept on the media for 48 h.

### Starvation resistance assay

For the starvation resistance assay, newly emerged male flies were maintained on standard food for about 7 days prior to the assay. Flies were then transferred to glass tubes (6.5 cm length×3 mm inner diameter) used with the *Drosophila* Activity Monitoring system (DAM, Trikinetics, MA, USA) containing 1% agar without any supplements. Since the circadian fluctuation of the starvation resistance has previously been reported [50], the starvation assay was initiated at ZT8 when the resistance to the starvation was reported to be maximal. Activity was monitored using the DAM system at 1 min intervals. Death of the flies was recognized by the cessation of locomotor activity and the number of deaths was counted in each hour. The assays were performed under 12∶12 light∶dark conditions at 25°C.

### Measurements of metabolic rate

Fly respirometry was performed using a hand-made respirometer made using a 1 ml plastic syringe attached to a 5 ml glass capillary, containing a small amount of soda lime (Wako, Osaka, Japan) behind a sponge placed in the body of the syringe. A single adult fly was placed in the syringe body and the plunger was inserted. After being placed on a flat surface at 25°C and allowed to equilibrate for 15 min, a small amount of ink was placed at the end of the capillary to create a closed metabolism chamber. The rate of movement of the ink was measured and the CO_2_ production was calculated [24]. A control was performed without a fly to correct for fluctuations in ambient temperature and air pressure. After respirometry, the fly was transferred to a 1.5 ml Eppendorf tube with oil, and the body weight of each fly was measured using a fine scale (Sartorius BP110S). The weight of control and *fmn* male flies were 1.54±0.33 (mean±SD) mg and 1.61±0.41 (mean±SD) mg, respectively. The value of the CO_2_ production normalized to body weight was calculated by dividing the volume of CO_2_ produced by the weight of the fly.

## Supporting Information

Movie S1 **Flies expressing** ***shi*** **^ts^ in motor neurons show paralysis with temperature increase.**
(MP4)
